# Influence of Surface Treatments and Thermal Aging Duration on the Shear Bond Strength of Resin Cement to CAD/CAM Monolithic Zirconia

**DOI:** 10.3390/polym18050592

**Published:** 2026-02-27

**Authors:** Etem Tayfun Gökşen, Ayşe Meşe, Tamer Akan

**Affiliations:** 1 Department of Prosthodontics, Faculty of Dentistry, Dicle University, Diyarbakır 21280, Türkiye; aysemese@yahoo.com; 2 Department of Physics, Faculty of Science, Eskisehir Osmangazi University, Eskisehir 26040, Türkiye; akan@ogu.edu.tr

**Keywords:** zirconia, cold plasma, sandblasting, femtosecond laser, thermal aging, resin cement, bond strength

## Abstract

This study aimed to evaluate the individual and combined effects of mechanical, plasma-based, and laser-based surface treatments, along with short- and long-term thermal aging, on the surface morphology, surface energy, and resin cement bond strength of CAD/CAM monolithic zirconia. Significant numerical differences were observed among the treatment groups. Surface roughness increased from 0.22 µm (control) to 0.98 µm after sandblasting, 1.12 µm after sandblasting + plasma, and 1.07 µm after laser treatment, while plasma alone produced a moderate increase (0.31 µm). Wettability improved most notably in the plasma group, where the contact angle decreased to 43.27° compared with 67.00° in the control. The highest shear bond strength after 5000 thermal cycles was recorded in the sandblasting + plasma group (14.80 ± 1.53 MPa), whereas laser treatment demonstrated the best long-term stability, showing no significant decrease after 10,000 cycles (12.48 → 12.02 MPa). From a practical perspective, these findings indicate that sandblasting followed by plasma treatment provides high initial bond strength, making it suitable for clinical situations requiring strong immediate adhesion of zirconia restorations. Conversely, femtosecond laser treatment offers superior resistance to aging-related degradation, suggesting its potential value in cases where long-term durability is critical, such as high-stress posterior restorations or patients with parafunctional habits.

## 1. Introduction

Zirconia, or zirconium oxide (ZrO_2_), is a polycrystalline ceramic material recognized for its excellent mechanical strength, biocompatibility, and favorable optical behavior, making it highly suitable for various restorative applications [1]. Its increasing use in dentistry is largely attributed to its metal-free composition, aesthetic advantages, and stable biocompatibility profile, which collectively enhance clinical acceptance [2]. Consequently, zirconia-based ceramics are now employed widely in crowns, fixed partial dentures, implant abutments, inlays, onlays, and full-contour restorations [3]. Monolithic zirconia restorations, particularly full-contour materials, are now preferred over traditional zirconia framework systems because of their high strength, fracture toughness and compatibility with digital production stages. Studies have shown that the transition from bilayered zirconia restorations (zirconia core with veneering porcelain) to monolithic zirconia systems eliminates the need for a porcelain veneer layer, thereby reducing technical complications such as veneer cracking and chipping. Additionally, CAD/CAM-based production workflows improve manufacturing efficiency in clinical practice [4,5].

Despite the increased efficiency, the absence of a silica phase and the inert surface morphology of zirconia restrict the effectiveness of bonding achieved through traditional acid etching and silane application. This disadvantageous clinical scenario means that achieving a permanent, aging-resistant bond with resin cement is critical for clinical success. Therefore, zirconia surface roughening using micromechanical (e.g., airborne-particle abrasion (APA)), chemical (e.g., 10-methacryloyloxydecyl dihydrogen phosphate (MDP)-containing primers/universal adhesives) or physical (e.g., tribochemical silica coating, plasma, or laser processes) treatments is recommended, either alone or in combination [6]. The subject of which surface treatments should be applied alone or in combination and what the application parameters should be remains under investigation, although it is not precisely known. Recent systematic reviews and current studies have reported that the application of APA + MDP stands out in terms of initial and post-aging bonding. In some cases, the tribochemical silica coating may also be advantageous [7].

In recent years, the application of femtosecond lasers (fs-lasers) to physical surface treatments has attracted attention. Current studies have shown that zirconia surface topography obtained using specific wavelengths and energy parameters with an fs laser yields shear bond strength (SBS) values similar to those obtained using APA; however, the efficiency of this method depends on the applied parameters [8,9,10]. These findings suggest that the fs laser could be an alternative to APA for zirconia surface treatment, but it has not yet been shown to consistently outperform APA [9]. Similarly, cold plasma (CP) treatment improves the surface energy and wettability by introducing polar oxygen-rich functional groups without causing structural damage, making it a minimally invasive alternative [11]. Current evidence indicates that CP treatment reduces the contact angle and enhances initial bond strength; however, its effectiveness is highly dependent on variables such as gas composition, power settings, exposure duration, and application sequence, and no consensus has yet been established regarding an optimal combination of these parameters [12].

Many studies have reported that the bond strength formed between zirconia and resin cement decreases significantly when artificial aging is applied, particularly following thermal cycling and long-term water storage [13,14,15]. Systematic reviews and experimental studies indicate that thermal cycling (i.e., repeated cycles between 5 and 55 °C) reduces shear bond strength and that prolonged exposure to water results in a further reduction due to hydrolysis and interfacial degradation. However, adhesive/primer-cement protocols containing 10-MDP can increase both the initial bond strength and its durability, and combinations of air abrasion and MDP-containing cement/primer can produce more stable results, even after aging. Furthermore, physical surface treatments that increase surface energy, such as the application of CP, can maintain bond values even after short-term thermal aging. However, the effectiveness of this system is sensitive to processing parameters (e.g., gas type and exposure time), and there is no full consensus on this in the literature [16,17,18].

In conclusion, the literature suggests that APA + MDP-containing protocols are still considered the “reference” approach, while methods such as fs laser and CP show promise when applied with the correct parameters. Demonstrating their durability under realistic aging conditions is crucial, as is studying the interactions between the material and the protocol in more detail. In addition to their physicochemical performance, these alternative surface treatments differ in terms of clinical accessibility, operational controllability, and practical feasibility in routine dental practice. CP systems are generally compact, chairside-applicable, and relatively cost-effective, with a limited number of adjustable parameters such as gas flow rate, power, and exposure time, facilitating integration into clinical workflows. In contrast, fs laser systems require sophisticated optical setups, precise beam delivery mechanisms, and strict control of parameters including pulse duration, fluence, repetition rate, and focal distance. Although this high level of control enables reproducible micro-structuring and potentially enhanced long-term stability, the elevated equipment cost, maintenance demands, and need for specialized training may restrict their routine clinical implementation. Taking these scientific and practical considerations into account, this study aimed to evaluate the comparative effects of different surface treatments and aging conditions on zirconia surface properties (e.g., roughness and contact angle) and resin cement bond strength.

The null hypothesis of this study was that neither the type of surface treatment nor the duration of thermal aging would significantly affect zirconia surface roughness, wettability (contact angle), or resin cement bond strength.

## 2. Materials and Methods

Ethical approval for this study was obtained from the Ethics Committee of the Faculty of Dentistry, Dicle University (ethical approval date: 24 April 2024, approval no: 2024-12).

The materials used in this study, along with their trade names, manufacturers, and chemical compositions, are presented in Table 1.

**Figure 1:**
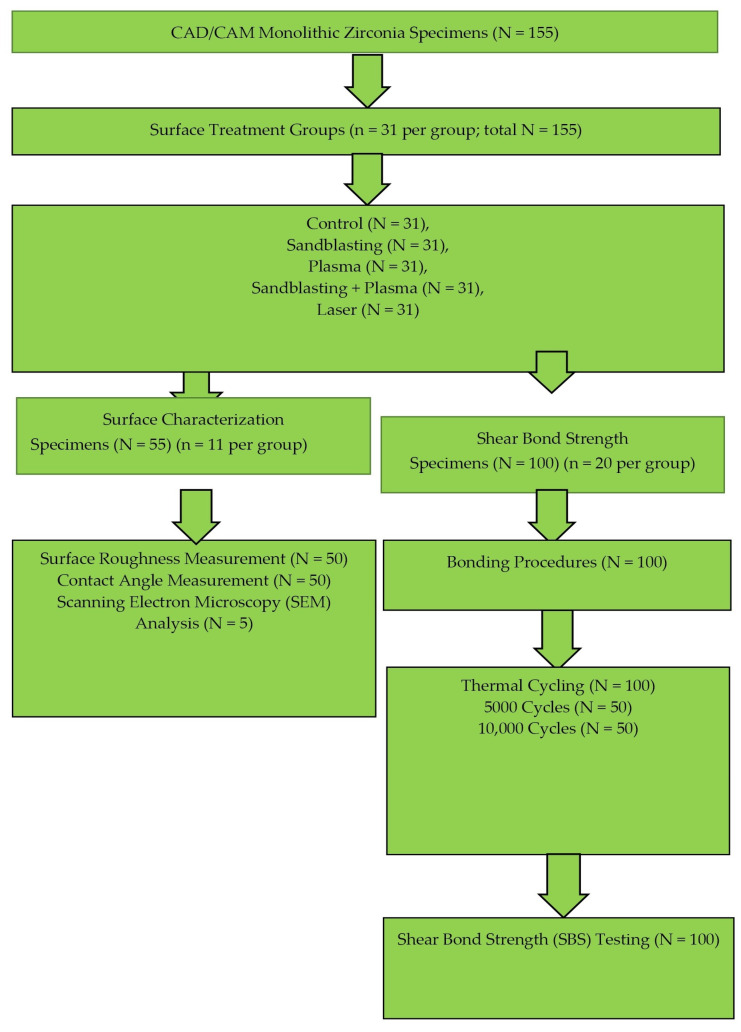
Flow chart of the experimental setup.

The workflow of the study is shown in Figure 1.

### 2.1. Specimen Preparation

This in vitro study was powered a priori using G*Power (v3.1) with an effect size of 0.40, a power (1–β) of 0.95, and α = 0.05, yielding a minimum sample size of *n* = 10 per subgroup [19,20]. Monolithic zirconia plates (12 × 15 × 2 mm) were designed in SolidWorks 2025 (Dassault Systèmes, Waltham, MA, USA), milled from 98 mm diameter, 20 mm height zirconia discs (Explore Esthetic D98 × 20; Upcera, Benxi, China) using a milling unit (Redon Hybrid, Istanbul, Türkiye), and sintered at 1480 °C for 8 h (Redon Rapid, Istanbul, Türkiye) according to the manufacturer’s instructions (final dimensions confirmed after ~20% sintering shrinkage). Each specimen was embedded in self-curing acrylic resin (18 × 18 × 18 mm molds; Imicryl, Konya, Türkiye), leaving the test surface exposed, sequentially polished with 400/600/1000-grit SiC papers, ultrasonically cleaned in saline for 10 min, and air-dried. A total of 155 specimens were randomly allocated to five main surface treatment groups (*n* = 31 per group): Control (C), Sandblasting (S), Plasma (P), Sandblasting + Plasma (SP), and Femtosecond Laser (L). Of these, 55 specimens (*n* = 11 per group) were used exclusively for surface characterization analyses, including SEM observation, surface roughness, and contact angle measurements. The remaining 100 specimens (*n* = 20 per group) were reserved for shear bond strength testing. Each group was further subdivided into two thermocycling subgroups (5000 or 10,000 cycles; *n* = 10 each), in accordance with the minimum sample size determined by statistical power analysis. This allocation ensured that surface characterization procedures did not influence the mechanical test results.

### 2.2. Surface Treatment

**Figure 2:**
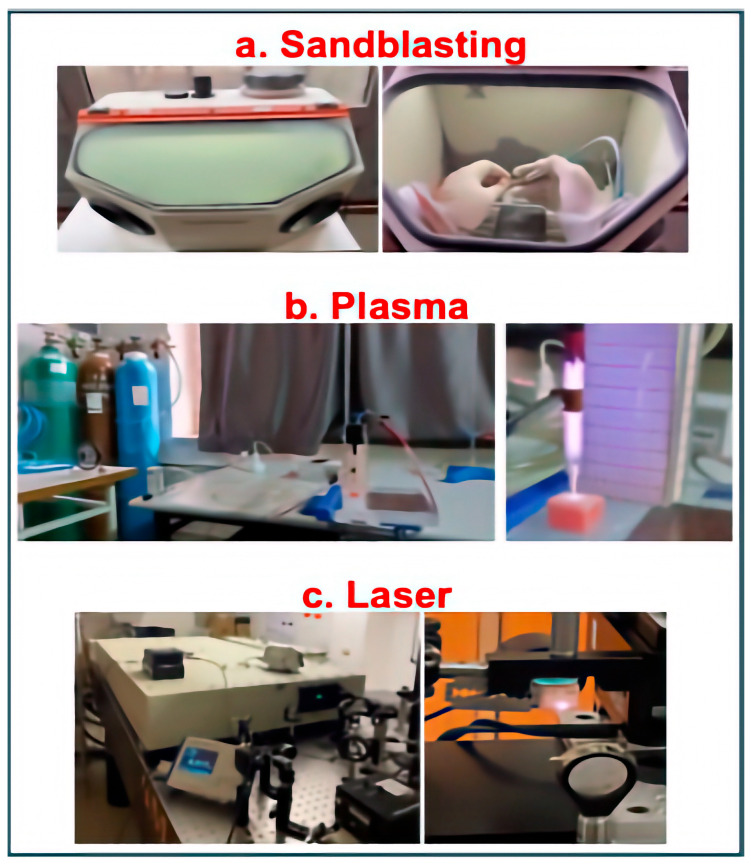
Representative photographs of the surface treatment procedures: (**a**) sandblasting with 50 µm Al_2_O_3_ particles, (**b**) application of cold argon plasma, and (**c**) surface ablation using a femtosecond laser system.

1. Control (C): No surface treatment was applied after sintering; specimens served as baseline controls.
2. Sandblasting (S): Airborne-particle abrasion (Figure 2a) was performed with 50 µm Al_2_O_3_ at 0.25 MPa, from ~10 mm, for 20 s (Dentsply International, Inc., Bohemia, NY, USA). Specimens were ultrasonically cleaned in distilled water for 10 min and air-dried.
3. Plasma (P): The zirconia samples were treated at the Cold Plasma Laboratory of Eskişehir Osmangazi University (Figure 2b). A custom coaxial plasma jet (quartz tube, central tungsten needle electrode, copper foil ground) was used with high-purity Argon (10 L/min); sinusoidal high voltage 6 kV_pp_ at 30 kHz. Specimens were placed 15 mm from the nozzle, and the bonding surface was scanned for 90 s at ambient temperature.
4. Sandblasting + Plasma (SP): Specimens underwent the sandblasting protocol, followed immediately by the plasma treatment.
5. Femtosecond Laser (L): Zirconia specimens were irradiated using a Ti:sapphire amplifier and an optical parametric system (Spectra Physics, Spitfire Pro XP, TOPAS, Milpitas, CA, USA) with a 50 fs pulse duration and a 1 kHz repetition rate (Figure 2c). The laser beam, operating at a central wavelength of 800 nm, was focused onto the specimens using a 10 cm biconvex lens, resulting in a measured beam radius (ω_0_) of 18 μm, as determined by the knife-edge technique. The ablation process was conducted at a power of 1.2 mW. The regulation of pulse fluence was achieved by means of an adjustment to the input laser energy via optical density filters, in conjunction with a precise control of the specimen’s position relative to the focal point of the lens, facilitated by LabVIEW software (Version 2025 Q1, National Instruments, Austin, TX, USA). During the treatment, the specimens were scanned at a speed of 1 mm/s using a computer-controlled XYZ translation stage to ensure a uniform surface topography.

### 2.3. Surface Characterization

#### 2.3.1. Surface Roughness Measurement

Surface roughness was measured for 10 specimens from each main group using a profilometer (Perthometer S2, Mahr, Göttingen, Germany). The measurement parameters were set to a tracing length of 1.75 mm, a cut-off length of 0.25 mm, and a stylus speed of 0.5 mm/s.

Three measurements were performed for each specimen, and the mean values were recorded. For each sample, three measurements were taken, and the average profile depth (R_a_) values were calculated.

#### 2.3.2. Contact Angle Measurement

Static water contact angle was measured with a video goniometer (Krüss GmbH, Hamburg, Germany) using a 3 µL distilled water droplet. Images were recorded at 10 s, with both sides averaged, and three measurements per specimen were averaged. A total of 10 specimens per group were used for contact angle measurements.

#### 2.3.3. Scanning Electron Microscopy (SEM) Analysis

One representative specimen per group was imaged at ×2000 magnification (Quanta 250 FEG, FEI, Eindhoven, The Netherlands) to examine topography relative to the control.

### 2.4. Bonding Procedures

A universal primer (Monobond Plus; Ivoclar Vivadent, Schaan, Liechtenstein) was applied for 60 s. PTFE molds (Ø 4 mm, height 3 mm) were used to define the bonding area. A dual-cure self-adhesive resin cement (Panavia SA Universal, Kuraray, Tokyo, Japan) was placed and light-cured for 20 s (O-Light LED unit, Woodpecker, Guilin, China), followed by removal of molds; specimens were stored in distilled water at 37 °C for 24 h before aging.

### 2.5. Thermal Cycling (Artificial Aging)

Specimens underwent 5000 or 10,000 cycles between 5 °C and 55 °C (dwell 30 s; transfer 5 s) using a thermocycler (Thermocycler 1100, SD Mechatronik, Feldkirchen-Westerham, Germany).

### 2.6. Shear Bond Strength (SBS) Testing

SBS was measured on a universal testing machine (Model 1082, Lloyd Instruments, Agawam, MA, USA) using a knife-edge technique at 90° to the interface and a crosshead speed of 1 mm/min, following ISO 29022:2013 [21]. Failure load (N) was divided by bonded area (12.56 mm^2^) to calculate MPa.

### 2.7. Statistical Analysis

The normality of continuous variables was examined using the Shapiro–Wilk test. Variables found to be normally distributed based on the normality test results were expressed as mean ± standard deviation, while variables not normally distributed were expressed using median (IQR) values. When normality was found in comparisons between the two groups, the independent two-sample *t*-test was used. Based on the normality test results, when there were more than two groups and normality was observed, the ANOVA test was used to compare the groups; when normality was not observed, the Kruskal–Wallis test was used. After the ANOVA test, subgroup analyses were performed using the Bonferroni test in cases of overall significance, and after the Kruskal–Wallis test, subgroup analyses were performed using the Dunn–Bonferroni test in cases of overall significance. SPSS (IBM Corp. Released 2017, IBM SPSS Statistics for Windows, Version 25.0. Armonk, NY, USA: IBM Corp.) was used for statistical analyses, and *p* < 0.05 was considered statistically significant.

## 3. Results

### 3.1. Surface Roughness Measurement

Surface roughness (R_a_) values were measured for all experimental groups to evaluate the effects of different surface treatments on the topographical characteristics of the CAD/CAM monolithic zirconia. Measurements were performed using a profilometer, and three readings from different regions of each specimen were averaged to obtain the final R_a_ value. Significant differences in surface roughness were observed among the groups (*p* < 0.001), indicating that each surface treatment produced distinct alterations on the zirconia surface. Table 2 summarizes the surface roughness (Ra, µm) and contact angle values of the tested groups.

Statistical analysis revealed a significant difference in the surface roughness among the five groups (*p* < 0.001). The Control group displayed the lowest roughness (0.22 ± 0.06 µm), indicating a smooth, untreated zirconia surface. In contrast, the Sandblasting + Plasma group exhibited the highest roughness (1.12 ± 0.15 µm), suggesting a strong synergistic effect between mechanical abrasion and plasma-induced surface activation. Both Sandblasting (0.98 ± 0.09 µm) and Laser treatment (1.07 ± 0.11 µm) produced substantial increases in surface roughness, forming irregular microretentive structures that enhance micromechanical interlocking. Meanwhile, Plasma alone produced a moderate yet noticeable increase (0.31 ± 0.07 µm), significantly lower than mechanically treated groups but slightly higher than the control group.

### 3.2. Contact Angle Measurement

**Figure 3:**
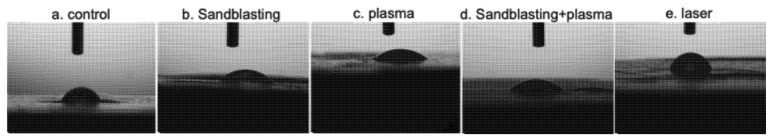
The photographs of the water-droplet images.

**Figure 4:**
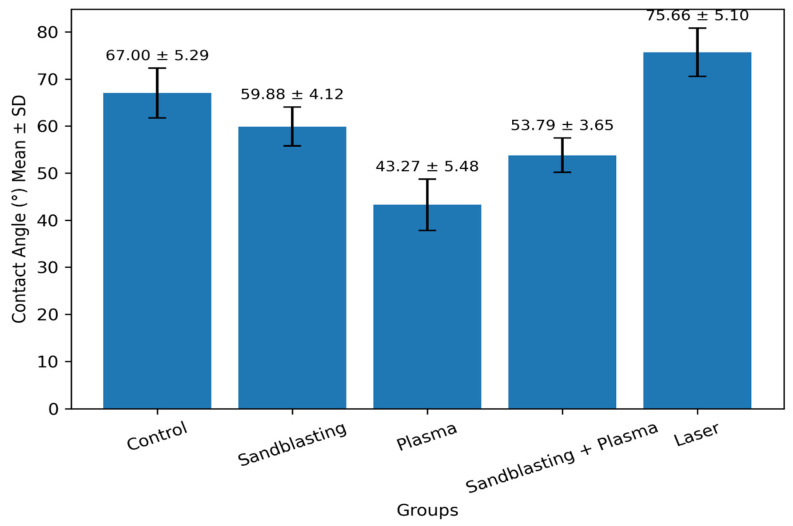
Contact angle values by treatment (mean ± SD).

Contact angle measurements were performed to evaluate how different surface treatments influence the wettability of zirconia. Representative water droplet contact angle images for each experimental group are presented in Figure 3, whereas the corresponding contact angle measurement values are provided in Table 2 (a: Control, b: Sandblasting, c: Plasma, d: Sandblasting + Plasma, e: Laser). The data demonstrated statistically significant differences among all groups, indicating that each treatment produced a unique alteration in surface wettability. The contact angle analysis revealed distinct differences among the surface treatment groups, as also illustrated in Figure 4. The Control group exhibited the highest wettability resistance with a mean contact angle of 67.00 ± 5.29°, whereas the Plasma group showed the lowest value at 43.27 ± 5.48°, indicating the greatest enhancement in surface wettability. Sandblasting produced a moderate reduction in the contact angle (59.88 ± 4.12°), while the combined Sandblasting + Plasma treatment further improved wettability, yielding a contact angle of 53.79 ± 3.65°. In contrast, the Laser group demonstrated the least favorable wettability, presenting the highest contact angle of 75.66 ± 5.10°, consistent with its more hydrophobic surface. These quantitative differences correspond directly with the graphical trends displayed in Figure 4, which visually compares the mean contact angle values across all five treatment conditions. Among the groups, Laser treatment produced the highest contact angle, reflecting the lowest surface wettability, and was significantly different from all other groups in pairwise comparisons. In contrast, the Plasma group showed the lowest contact angle, corresponding to the highest wettability, and differed significantly from every other treatment (*p* < 0.001).

The Control group exhibited significantly higher contact angles than Plasma, Sandblasting, Laser, and Sandblasting + Plasma groups. Sandblasting alone resulted in a lower contact angle than Laser (*p* < 0.001) but a higher angle compared with the Sandblasting + Plasma group. Overall, pairwise statistical analysis confirmed significant differences across all surface treatments, with Laser consistently yielding the highest contact angle and Plasma the lowest. The combined Sandblasting + Plasma protocol further enhanced wettability relative to Sandblasting alone, while the Control surface remained significantly less wettable than Plasma and Sandblasting + Plasma.

### 3.3. SEM Images Analysis

**Figure 5:**
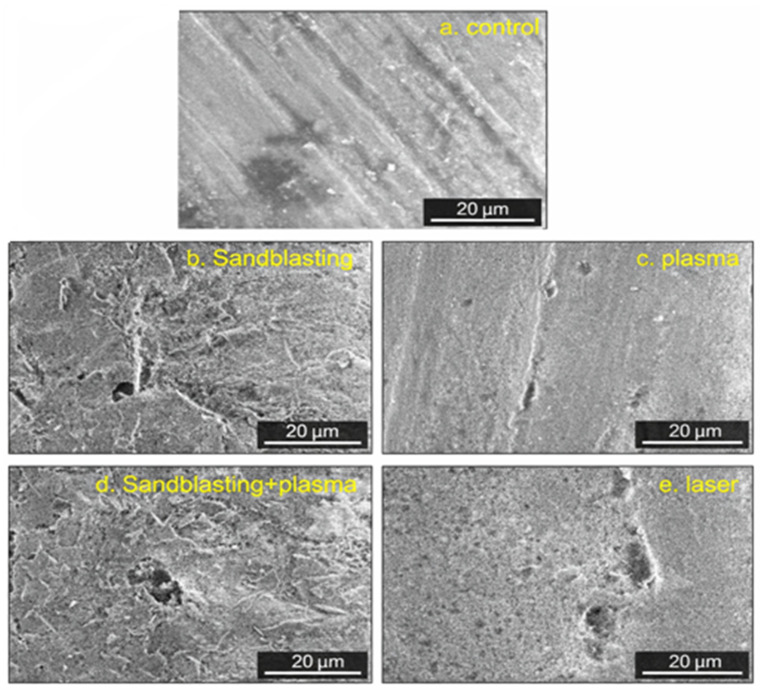
Representative scanning electron microscopy (SEM) images of the surface treatments (magnification ×2000, scale bar = 20 µm). (**a**) Control group; (**b**) Sandblasting group; (**c**) Plasma group; (**d**) Sandblasting + Plasma group; (**e**) Laser group.

Figure 5 presents the SEM images of the zirconia surfaces, where (a) shows the untreated control specimen, (b) the sandblasted surface, (c) the plasma-treated surface, (d) the sandblasting followed by plasma treatment, and (e) the femtosecond laser-treated surface.

The SEM analysis revealed significant morphological differences among the surface treatments compared to the control group. While the control group (a) was characterized by a surface with low roughness and limited micromechanical features, the experimental groups exhibited distinct topographical alterations. In the sandblasting group (b), the particle abrasion process created evident “peak-and-valley” formations and micro-craters. This aggressive modification significantly increased the surface area, providing the necessary irregularity for micromechanical retention. Conversely, the plasma group (c) displayed a topography with smoother transitions and greater homogeneity despite an increase in roughness. This suggests that plasma treatment primarily modifies the surface energy and cleanliness, thereby enhancing wettability potential rather than creating deep mechanical undercuts. The laser-treated group (e) presented a heterogeneous roughness profile characterized by signs of thermal ablation, including melting and re-solidification tracks and wavy structures. However, the combined sandblasting and plasma group exhibited a morphology that appeared more favorable for micromechanical interlocking and chemical bonding. (d). In this group, the retentive micro-structures generated by sandblasting were preserved, while the subsequent plasma application likely cleaned and homogenized the surface. This synergistic effect created a surface topography that is highly favorable for both micromechanical interlocking and chemical bonding.

### 3.4. Shear Bond Strength Findings

Table 3 illustrates the shear bond strength values following 5000 and 10,000 thermal cycling procedures, providing a comparative evaluation of the effects of short- and long-term artificial aging.

The comparative evaluation of shear bond strength (SBS) among the various surface-conditioning methods demonstrated that the Sandblasting + Plasma group exhibited the highest bonding performance after 5000 thermal cycles (14.80 ± 1.53 MPa). This outcome reflects a pronounced synergistic interaction between the two treatments: sandblasting introduces a retentive and topographically complex surface characterized by micro-irregular “peak-and-valley” formations, while plasma treatment enhances superficial cleanliness, increases surface energy, and promotes chemical homogeneity. Collectively, these morphological and physicochemical modifications create an optimized substrate for both micromechanical interlocking and chemical adhesion mechanisms. Conversely, the Control group presented the lowest SBS values (2.73 ± 0.87 MPa), significantly inferior to all treated groups (*p* < 0.001), underscoring the inadequacy of untreated, low-roughness zirconia surfaces for achieving clinically reliable bonding. The influence of extended thermal aging (10,000 cycles) further elucidated the durability characteristics of each treatment modality. Although Sandblasting initially produced high bond strength (12.73 ± 1.03 MPa), it experienced a statistically significant reduction to 10.49 ± 0.90 MPa upon aging, indicating that mechanically roughened interfaces may undergo hydrothermal degradation over time. A similar trend was observed in the Plasma group, where SBS values decreased significantly (*p* = 0.036). This suggests that the benefits conferred by plasma-induced surface activation—primarily increases in wettability and superficial chemical reactivity—may be susceptible to deterioration under repeated thermal cycling.

Of particular note, the Laser treatment group demonstrated the most pronounced resistance to thermal aging. Despite having initial SBS values (12.48 ± 1.59 MPa) comparable to those of the sandblasting-based groups, the laser-treated specimens exhibited no statistically significant decline after 10,000 cycles (*p* = 0.511). SEM analyses indicating localized melting, re-solidification, and the formation of wave-like surface textures suggest that laser irradiation produces a distinct microtopography with enhanced structural endurance under thermal fatigue. When considered together, these findings imply that the Sandblasting + Plasma combination maximizes initial bonding potential, whereas laser conditioning offers superior long-term stability, thereby presenting two complementary strategies for optimizing zirconia bonding performance depending on the clinical demand for either maximal immediate adhesion or enhanced aging resistance.

## 4. Discussion

Zirconia is frequently preferred in dental applications because of its high mechanical durability and ideal biocompatibility. However, one of the limitations in the use of zirconia ceramics is their chemically inert surface morphology and lack of mechanical interlocking. Consequently, they cannot achieve a strong bond with dental adhesive resin cements [22]. To overcome this bonding issue, various surface treatments—classified as mechanical, thermal, and chemical methods—have been investigated in studies to modify surface properties and increase shear bond strength (SBS) [23]. In this study, the effects of femtosecond (FS) laser, cold plasma, and sandblasting surface treatments—which can be applied to strengthen the zirconia–resin cement bond—were examined. Additionally, to evaluate the change in bond strength over time following these treatments, samples were subjected to a two-stage thermal aging procedure (short-term and long-term). The findings of this study indicate that different surface treatments affect the shear bond strength of zirconia material differently. Based on the results obtained, the null hypothesis was rejected.

Surface roughening with hydrofluoric (HF) acid was not preferred in this study because numerous previous studies have reported that HF acid is ineffective on silica-free, polycrystalline zirconia [24,25,26]. A review of the literature shows that sandblasting is a fundamental technique for increasing the bond strength between zirconia and resin cement. It has been reported that the use of different Al_2_O_3_ particle sizes in sandblasting creates abrasion that appears as distinct micro-retentive structures; simultaneously, it improves the penetration of the resin cement by increasing surface energy, thereby providing a strong effect on bonding [27]. Furthermore, it has been shown that sandblasting pressure and application distance directly affect surface morphology just as particle size does. While sandblasting with ideal values increases bond strength, overly aggressive conditions increase the tetragonal-to-monoclinic phase transformation, leading to stress formation and failure in long-term stability [9]. It has been reported that the roughness resulting from surface treatments increases bond strength [28]. This increase in roughness strengthens the micromechanical bonding between zirconia and resin cement because the expansion of the contact area increases the penetration of resin infiltration into the surface [29]. In this study, the bond strength values of the sandblasted group increased significantly, aligning with the literature.

Non-thermal atmospheric plasma has been studied in the literature [30] as an alternative to sandblasting. Studies have demonstrated that this is an effective method for increasing the bond strength at the zirconia–resin cement interfaces. It has been reported [31,32] that plasma application causes chemical effects—rather than mechanical ones—by increasing the oxygen content and hydroxyl groups on the zirconia surface. These chemical effects lead to an increase in the surface energy and, consequently, stronger wettability. The same studies highlight that since plasma operates at low temperatures, it does not cause undesirable structural changes, such as phase transformation, micro-crack formation, or micro-abrasion, offering a minimally invasive advantage. Similar to these, other studies [33,34] have stated that plasma application increased the bond strength between zirconia and resin cement. Consistent with the literature, the zirconia–resin cement bond strength values in our study showed an increase compared to the control group.

The application of plasma after sandblasting significantly improves bond strength by increasing both mechanical and chemical activation at the zirconia–resin cement interface [34], creating a synergistic surface modification. Sandblasting roughens the zirconia surface topography at the micro-scale to allow resin penetration and increase surface energy. Subsequently, plasma application increases surface oxygenation and raises the polar component, enhancing the hydrophilicity and wettability of the surface [33]. In a study by Kim et al. [32], although bond strength values in groups receiving plasma after sandblasting were initially higher than those in the sandblasting-only group, it was noted that after thermal aging, these values were lower than those in the sandblasting group. Conversely, another study [35] examining the effect of sandblasting and plasma on bonding reported that plasma application after sandblasting increased bond strength. Similarly, another study [36] reported that argon plasma application after sandblasting significantly increased both the surface energy of zirconia and the bond strength and durability with resin cement. It was stated that after CP treatment, surface hydrophilicity increased significantly while surface morphology remained unchanged. The combination of sandblasting and plasma has been shown to significantly increase bonding values between zirconia and resin cement by increasing roughness [37]. In this study, it was observed that plasma application after sandblasting led to a decreased contact angle and resulted in higher bond strength than the sandblasting-only group.

It has been reported in the literature that fs laser ablation creates a more homogeneous and easily controlled micro-roughness on the zirconia surface compared to other laser types, minimizing thermal damage, micro-fractures, and phase transformation [38]. It should be noted that although the literature reports that femtosecond laser treatment may minimize thermal damage and limit tetragonal–monoclinic phase transformation, this interpretation in the present study is indirect because no crystallographic analysis (such as XRD) was conducted to directly assess potential phase changes. Due to the ultra-short pulses (~10^−15^ s) of fs laser application, it is stated that only the targeted micro-matrix is affected, thereby protecting adjacent tissues [39]. Thus, it is reported to provide high mechanical and chemical bonding surfaces on zirconia. Studies [40,41,42,43] have reported that femtosecond laser application on high-translucency zirconia ceramics increases shear bond strength values when bonded with resin cement. The results of this study closely overlap with findings where FL application on zirconia increased surface roughness, contact angle, and bonding values compared to the control group [44,45,46]. This unexpected increase in contact angle is thought to be related to the direction of the grooves on the surface after laser treatment. The shear bond strength values found in this study, which were similar to the sandblasting treatment, suggest that it could be used as an alternative surface treatment.

In this study, the effect of thermal aging on bond strength was found to be surface treatment dependent. As seen in the sandblasting, plasma, and sandblasting + plasma groups (*p* < 0.05), thermal cycle application in the literature studies generally significantly reduced the shear bond strength between the zirconia materials and self-adhesive resin cements used [16,47,48]. In contrast, the difference in bond strength between 5000 and 10,000 thermal cycles was not statistically significant in the control and laser groups (*p* > 0.05). Therefore, our hypothesis that “different thermal aging durations do not affect bond strength” was generally rejected but could not be rejected for the control and laser groups. This finding indicates that thermal stresses can negatively affect bonding, especially on zirconia surfaces that have undergone mechanical/chemical surface modification, whereas laser-treated surfaces remain relatively stable against thermal changes. This result suggests that fs laser application may demonstrate a more successful bond strength in the long term.

Limitations of this study include the use of a single high-translucency zirconia material, a single resin cement and primer system, the evaluation of only one parameter set for plasma and femtosecond laser applications, and the absence of comparison with other recommended surface treatments. Although this design allowed standardization of experimental conditions, the generalizability of the findings is limited. Zirconia systems with different yttria contents (e.g., 3Y-TZP, 4Y-PSZ, 5Y-PSZ, and 6Y-PSZ) exhibit distinct phase compositions and transformation behaviors that may influence surface reactivity and bonding performance. In addition, aging was limited to thermal cycling, and bond strength was assessed using static mechanical testing only. Future studies should investigate different zirconia compositions, broader aging protocols (including long-term water storage and cyclic fatigue), crystallographic phase analysis (e.g., XRD), failure mode evaluation, and diverse surface treatment parameters to provide a more comprehensive understanding of long-term zirconia–resin cement adhesion. Another limitation is the use of a universal primer in all groups without a no-primer control. Although this approach reflects clinically relevant bonding protocols, it prevents independent assessment of the relative contributions of surface treatment and chemical priming to bond strength. Future studies using factorial designs with primer and no-primer conditions are needed to clarify their individual and combined effects on zirconia–resin cement adhesion. Failure mode analysis was not systematically performed. Although shear bond strength testing provides quantitative data, it does not fully characterize the nature of interfacial adhesion. The absence of failure classification limits interpretation of whether bond degradation was interfacial or material-related. Future studies should incorporate qualitative and quantitative failure mode analysis for a more comprehensive evaluation of bonding performance. The aging protocol was limited to thermal cycling, which does not fully replicate the multifactorial conditions of the oral environment. The absence of long-term water storage and mechanical fatigue loading restricts extrapolation to long-term clinical performance. Future studies should incorporate extended aging and dynamic fatigue protocols to better simulate oral service conditions.

## 5. Conclusions

Based on these findings, the combination of sandblasting and plasma treatment showed promising results for achieving high initial bond strength, while femtosecond laser treatment showed potential for preserving long-term bond strength.

## Figures and Tables

**Table 1:** Materials used in this study, their places of manufacture, and their compositions.

Materials	Trade Name	Manufacturer	Composition		Lot No.	Expiry Data
Zirconia ceramics	Upcera Explore Esthetic	Shenzhen Upcera Co., Ltd., Shenzhen, China	ZrO_2_ + HfO_2_	86.3–94.2%	L2230309004-032	7 March 2028
			Y_2_O_3_	5.8–9.7%		
			Al_2_O_3_	<0.5%		
			Fe_2_O_3_	<0.5%		
			Er_2_O_3_	<2.0%		
			Other oxides	<0.5%		
Alumina oxide particle	Renfert 50	Renfert 50; Strahlmittel, Germany	Al_2_O_3_		15941112	13 October 2026
Adhesive resin luting agent	Panavia SA Cement Plus	Kuraray (Hattersheim, Germany)	(Paste A) contains 10-methacryloyloxydecyl dihydrogen phosphate monomer, Bis-GMA, TEGDMA, HEMA, silanated barium glass filler, silanated colloidal silica, dl-camphorquinone, peroxide, catalysts, and pigments, while the catalyst (Paste B) has hydrophobic aromatic dimethacrylate, silane coupling agent, silanated barium glass filler, aluminum oxide filler, surface-treated sodium fluoride, dl-camphorquinone, accelerators, and pigments		2J0409	31 December 2027
Ceramic primer	Monobond Plus	Ivoclar Vivadent AG, Schaan, Liechtenstein	Silane methacrylate, phosphoric acid methacrylate, ethanol, sulfide methacrylate		Z07R3T	19 November 2026
Acrylic resin	SC Acrylıc repair material	Imicryl Dental, Konya, Türkiye	Polymethyl methacrylate (PMMA), methyl methacrylate (MMA)		B270	10 October 2026

**Table 2:** The mean ± standard deviation values for surface roughness (µm) and contact angle (°) are shown for each experimental group.

Group	Surface Roughness (µm)	Contact Angle (°)		
Control	0.22 ± 0.06	67.00 ± 5.29		
Sandblasting	0.98 ± 0.09	59.88 ± 4.12		
Plasma	0.31 ± 0.07	43.27 ± 5.48		
Sandblasting + Plasma	1.12 ± 0.15	53.79 ± 3.65		
Laser	1.07 ± 0.11	75.66 ± 5.10		
			*p* Value * Surface Roughness	*p* Value ** Contact Angle
Control–Sandblasting			*p* < 0.05	*p* < 0.05
Control–Plasma			*p* < 0.05	*p* < 0.05
Control–Sandblasting + Plasma			*p* < 0.05	*p* < 0.05
Control–Laser			*p* < 0.05	*p* < 0.05
Sandblasting–Plasma			*p* < 0.05	*p* < 0.05
Sandblasting–Sandblasting + Plasma			*p* < 0.05	*p* < 0.05
Sandblasting–Laser			NS	*p* < 0.05
Plasma–Sandblasting + Plasma			*p* < 0.05	*p* < 0.05
Plasma–Laser			*p* < 0.05	*p* < 0.05
Laser–Sandblasting + Plasma			NS	*p* < 0.05

Data are presented as mean ± standard deviation. *p* < 0.05 was considered statistically significant, while NS indicates non-significant differences (*p* ≥ 0.05). Statistical comparisons were performed using the Kruskal–Wallis test or one-way ANOVA, as appropriate. *: Kruskal–Wallis test. **: ANOVA test.

**Table 3:** Shear bond strength (SBS) values (MPa) of zirconia ceramics subjected to different surface treatments and thermal aging protocols.

Group	SBS (MPa) (Mean ± SD)		*p* Value ***	
	5000 Thermocycles	10,000 Thermocycles		
Control	2.73 ± 0.87	2.45 ± 0.59	NS	
Sandblasting	12.73 ± 1.03	10.49 ± 0.90	*p* < 0.05	
Plasma	6.19 ± 0.84	5.31 ± 0.87	*p* < 0.05	
Sandblasting + Plasma	14.80 ± 1.53	13.19 ± 1.21	*p* < 0.05	
Laser	12.48 ± 1.59	12.02 ± 1.43	NS	
			*p* Value **	*p* Value **
Control–Sandblasting			*p* < 0.05	*p* < 0.05
Control–Plasma			*p* < 0.05	*p* < 0.05
Control–Sandblasting + Plasma			*p* < 0.05	*p* < 0.05
Control–Laser			*p* < 0.05	*p* < 0.05
Sandblasting–Plasma			*p* < 0.05	*p* < 0.05
Sandblasting–Sandblasting + Plasma			*p* < 0.05	*p* < 0.05
Sandblasting–Laser			NS	*p* < 0.05
Plasma–Sandblasting + Plasma			*p* < 0.05	*p* < 0.05
Plasma–Laser			*p* < 0.05	*p* < 0.05
Laser–Sandblasting + Plasma			*p* < 0.05	NS

Data are presented as mean ± standard deviation. *p* < 0.05 was considered statistically significant, while NS indicates non-significant differences (*p* ≥ 0.05). ***: Independent Samples *t* test. **: ANOVA test.

## Data Availability

The original contributions presented in this study are included in the article. Further inquiries can be directed to the corresponding author.
